# 4-Styryl­quino­lines from cyclo­condensation reactions between (2-amino­phen­yl)chalcones and 1,3-diketones: crystal structures and regiochemistry

**DOI:** 10.1107/S2053229620010803

**Published:** 2020-08-13

**Authors:** Diego Rodríguez, Sergio Andrés Guerrero, Alirio Palma, Justo Cobo, Christopher Glidewell

**Affiliations:** a Laboratorio de Síntesis Orgánica, Escuela de Química, Universidad Industrial de Santander, AA 678, Bucaramanga, Colombia; b Departamento de Química Inorgánica y Orgánica, Universidad de Jaén, 23071 Jaén, Spain; c School of Chemistry, University of St Andrews, Fife KY16 9ST, Scotland

**Keywords:** synthesis, cyclo­condensation, quinolone, crystal structure, mol­ecular conformation, hydrogen bonding, supra­molecular assembly, regiochemistry

## Abstract

Structures are reported for two matched sets of substituted 4-styryl­quino­lines which were prepared by the formation of the heterocyclic ring in cyclo­condensation reactions between 1-(2-amino­phen­yl)-3-aryl­prop-2-en-1-ones with 1,3-dicarbonyl com­pounds. (*E*)-3-Acetyl-4-[2-(4-meth­oxy­phen­yl)ethen­yl]-2-methyl­quino­line, C_21_H_19_NO_2_, (I), (*E*)-3-acetyl-4-[2-(4-bromo­phen­yl)ethen­yl]-2-methyl­quino­line, C_20_H_16_BrNO, (II), and (*E*)-3-acetyl-2-methyl-4-{2-[4-(tri­fluoro­meth­yl)phen­yl]ethen­yl}quino­line, C_21_H_16_F_3_NO, (III), are isomorphous and in each structure the mol­ecules are linked by a single C—H⋯O hydrogen bond to form *C*(6) chains. In (I), but not in (II) or (III), this is augmented by a C—H⋯π(arene) hydrogen bond to form a chain of rings; hence, (I)–(III) are not strictly isostructural. By contrast with (I)–(III), no two of ethyl (*E*)-4-[2-(4-meth­oxy­phen­yl)ethen­yl]-2-methyl­quino­line-3-carboxyl­ate, C_22_H_21_NO_3_, (IV), ethyl (*E*)-4-[2-(4-bromo­phen­yl)ethen­yl]-2-methyl­quino­line-3-carboxyl­ate, C_21_H_18_BrNO_2_, (V), and ethyl (*E*)-2-methyl-4-{2-[4-(tri­fluoro­meth­yl)phen­yl]ethen­yl}quino­line-3-carboxyl­ate, C_22_H_18_F_3_NO_2_, (VI), are isomorphous. The mol­ecules of (IV) are linked by a single C—H⋯O hydrogen bond to form *C*(13) chains, but cyclic centrosymmetric dimers are formed in both (V) and (VI). The dimer in (V) contains a C—H⋯π(pyrid­yl) hydrogen bond, while that in (VI) contains two independent C—H⋯O hydrogen bonds. Comparisons are made with some related structures, and both the regiochemistry and the mechanism of the heterocyclic ring formation are discussed.

## Abstract 2

Structures are reported for two sets of substituted 4-styryl­quino­lines formed by reactions of (2-amino­phen­yl)chalcones either with pentane-2,4-dione or, regiospecifically, with ethyl 3-oxo­butano­ate.

## Introduction   

**Figure 1:**
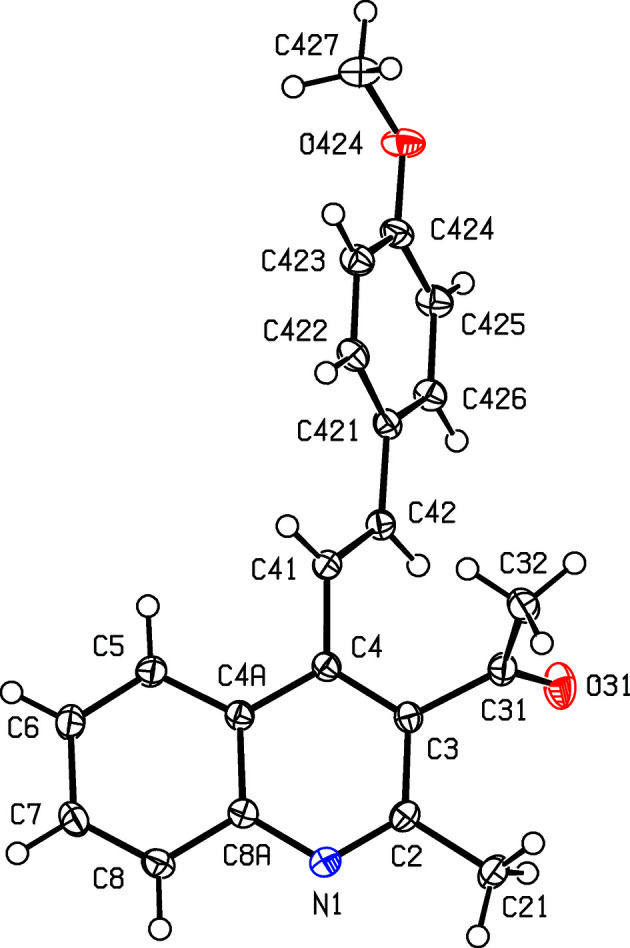
The mol­ecular structure of com­pound (I), showing the atom-labelling scheme. Displacement ellipsoids are drawn at the 50% probability level.

**Figure 4:**
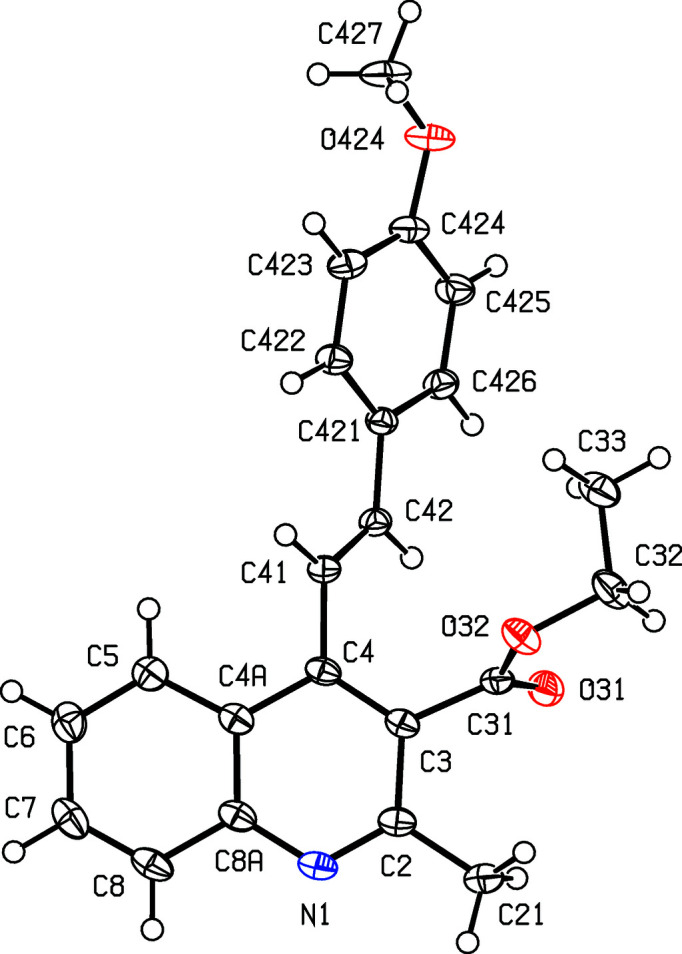
The mol­ecular structure of com­pound (IV), showing the atom-labelling scheme. Displacement ellipsoids are drawn at the 50% probability level.

Compounds containing 2-styryl­quino­line units have attracted inter­est in recent years because of their potential as anti­cancer (El-Sayed *et al.*, 2018 ▸), anti-HIV (Polanski *et al.*, 2002 ▸), anti­malarial (Roberts *et al.*, 2017 ▸) and anti­microbial (Cieslik *et al.*, 2012 ▸) agents, as well as in the treatment of Alzheimer’s dementia (Wang *et al.*, 2015 ▸). By contrast, analogous com­pounds containing 4-styryl units have been very much less extensively investigated, probably, at least in part, because of a lack of efficient and versatile methods for their synthesis: such methods have generally been based on coupling reactions requiring the prior synthesis of halo­quino­lines or (haloalk­yl)quino­lines, combined with either harsh reaction conditions or the use of expensive heavy-metal catalysts (Omar & Hormi, 2009 ▸; Xia *et al.*, 2016 ▸). However, a very straightforward synthesis of 4-styryl­quino­lines has been developed recently (Meléndez *et al.*, 2020 ▸), in which the heterocyclic ring of the quino­line unit is built *in situ* using a cyclo­condensation reaction between a 2′-amino­chalcone, (*A*), and a 1,3-dicarbonyl com­pound (*cf*. Scheme 1). The chalcone com­ponent in this type of cyclization is readily accessible by reaction between 2′-amino­aceto­phenone and an aromatic aldehyde, allowing incorporation of a wide variety of substituents both in the styryl portion and at the 3-position of the quino­line nucleus. We report here the mol­ecular structures and supra­molecular assembly of two matched sets, each of three related products: the 3-acetyl derivatives, com­pounds (I)–(III) (Scheme 1 and Figs. 1 ▸–3 ▸ ▸), where *X* = Me, were all obtained using pentane-2,4-dione as the dicarbonyl com­ponent, while the 3-carboeth­oxy derivatives, com­pounds (IV)–(VI) (Figs. 4 ▸–6 ▸ ▸), where *X* = OEt, were all obtained using ethyl 3-oxo­butano­ate (ethyl aceto­acetate). Compounds such as (I)–(III), containing an acetyl 
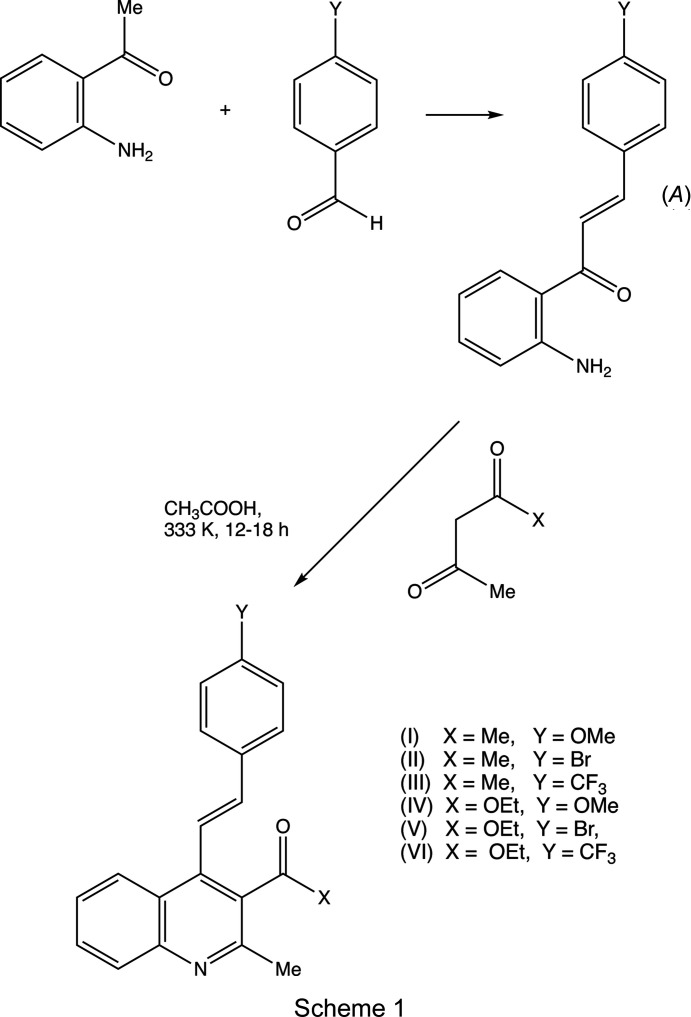
group, can act as useful synthetic inter­mediates, as they can undergo condensation with a further substituted aldehyde to form a chalcone substituent at the 3-position, as exemplified by com­pound (VIII) (Scheme 2). Such chalcones can themselves then undergo cyclo­condensation reactions, for example, with a hydrazine, to form either a pyrazole, under basic conditions (Samshuddin *et al.*, 2014 ▸), or a reduced pyrazole ring, under acidic conditions (Jasinski *et al.*, 2010 ▸), or with guanidine to form a reduced pyrimidine ring (Nayak *et al.*, 2014 ▸), thus giving access to a rich diversity of new 4-styryl­quinolin-3-yl heterocycles. In addition to reporting the mol­ecular and supra­molecular structures of com­pounds (I)–(VI), we also briefly consider com­pounds (VII) and (VIII) (Scheme 2). These have been reported on a simple proof of constitution basis [Cambridge Structural Database (CSD; Groom *et al.*, 2016 ▸) refcodes MUMZEC and MUMZIG (Meléndez *et al.*, 2020 ▸)] but without any structure description or discussion; accordingly, we discuss here the supra­molecular assembly in these two com­pounds.
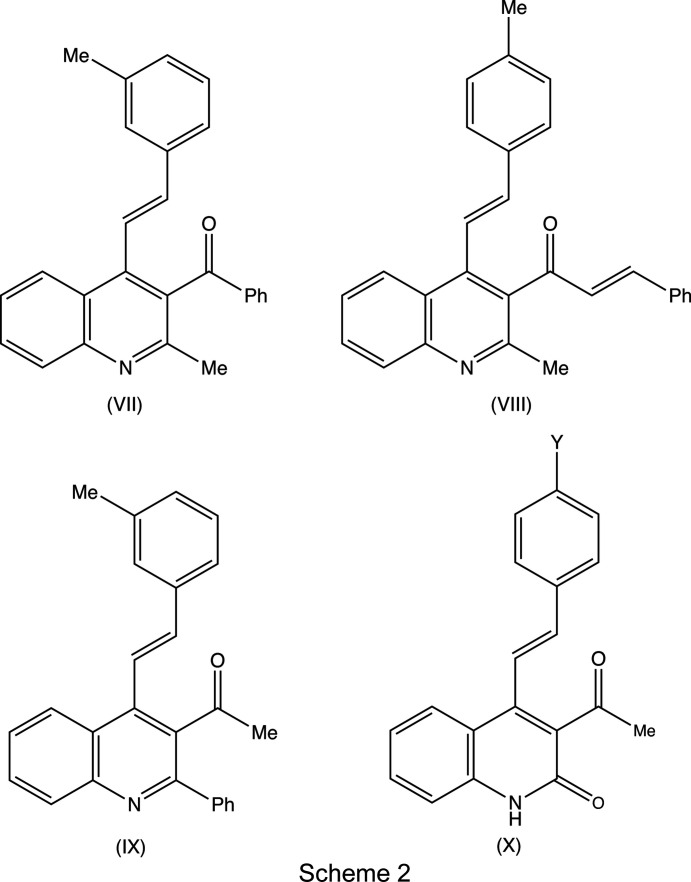


## Experimental   

### Synthesis and crystallization   

Samples of com­pounds (I)–(VI) were prepared and crystallized following a recently published procedure (Meléndez *et al.*, 2020 ▸).

### Refinement   

Crystal data, data collection and structure refinement details for com­pounds (I)–(VI) are summarized in Table 1 ▸. Two low-angle reflections which had been attenuated by the beam stop [100 for (I) and 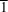
01 for (VI)] were omitted from the data sets before the final refinements; likewise, two bad outlier reflections (639 and 606) were removed from the data set for (IV). All H atoms were located in difference maps and subsequently treated as riding atoms in geometrically idealized positions, with C—H = 0.95 (alkenyl, aromatic and heteroaromatic), 0.98 (CH_3_) or 0.99 Å (CH_2_), and with *U*_iso_(H) = *kU*_eq_(C), where *k* = 1.5 for the methyl groups, which were permitted to rotate but not to tilt, and 1.2 for all other H atoms. For com­pounds (VII) and (VIII), the published structures (Meléndez *et al.*, 2020 ▸) were inverted and the atom labelling adjusted slightly in order to bring them into full conformity with com­pounds (I)–(VI) (*cf*. Tables 2 ▸ and 3 ▸); the modified versions of the CIFs for (VII) and (VIII) are provided in the supporting information. Examination of the structure for (VIII) using *PLATON* (Spek, 2020 ▸) showed that the unit cell contains two voids, each of volume 60 Å^3^ and centred at (0, 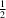, 0) and (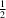, 0, 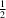), but further examination using SQUEEZE (Spek, 2015 ▸) showed that these voids contained negligible electron density.

## Results and discussion   

In reactions between a chalcone of type (*A*) (Scheme 1) and a symmetrical 1,3-diketone, such as pentane-2,4-dione, only a single product is possible, namely, the 3-acetyl-2-methyl­quino­line derivative, as exemplified by com­pounds (I)–(III). However, a com­parable reaction involving an unsymmetrical diketone, such as 1-phenyl­butane-1,3-dione can give two regioisomers, such as (VII), if the amino group reacts at the acetyl carbonyl group, or the alternative (IX) if the reaction occurs at the benzoyl carbonyl group. In general, reactions with this ketone lead exclusively to the 3-benzoyl-2-methyl isomers, as exemplified by (VII), rather than to the 3-acetyl-2-phenyl alternative exemplified by (IX) (Meléndez *et al.*, 2020 ▸), which is consistent with the greater reactivity in the nucleophilic addition reaction of acetyl groups com­pared with benzoyl groups (Bürgi *et al.*, 1974 ▸; Katritzsky *et al.*, 1995 ▸; Meléndez *et al.*, 2020 ▸). Similarly, the reaction of a chalcone of type (*A*) with an unsymmetrical diketo com­pound, such as ethyl 3-oxo­butano­ate, can, in principle, give two types of product: reaction of the amino group at the acetyl carbonyl group leads to ethyl esters, as exemplified by com­pounds (IV)–(VI), but reaction of the amino group at the ester carbonyl group would lead to elimination of ethanol with the formation of a 2-quinolone of type (X) (Scheme 2). Again, these reactions appear to lead exclusively to the esters, as exemplified by (IV)–(VI) (Meléndez *et al.*, 2020 ▸), consistent with the greater electrophilicity of a ketonic carbonyl group com­pared with an ester carbonyl group. On the other hand, 2-aryl-4-quinolones are sometimes formed as by-products arising from an intra­molecular cyclization of the chalcone precursor.

Compounds (I)–(III), where *X* = Me and *Y* = OMe, Br or CF_3_, respectively (Scheme 1 and Figs. 1 ▸–3 ▸ ▸), all crystallize in the space group *P*2_1_/*c* with rather similar unit-cell dimensions (Table 1 ▸) and very similar mol­ecular conformations (Table 2 ▸); each structure can be refined using the coordinates of one of the others as the starting point, provided due alteration is made in the substituent at atom C424 (Figs. 1 ▸–3 ▸ ▸). However, although there are short inter­molecular C—H⋯O and C—H⋯π(arene) contacts in all three com­pounds, involving the same sets of atoms (Table 3 ▸), in each of com­pounds (II) and (III), the H⋯*Cg* distance is quite long and probably of marginal structural significance, whereas it can be regarded as a genuine hydrogen bond in com­pound (I). On this basis, com­pounds (I)–(III) can be regarded as isomorphous but not strictly isostructural (Acosta *et al.*, 2009 ▸; Blanco *et al.*, 2012 ▸). However, in the com­parable series of com­pounds, *i.e.* (IV)–(VI), where *X* = OEt, although com­pounds (IV) and (VI) are both triclinic, in (IV) the inter-axial angles are all less than 90°, but in (VI) they are greater than 90°, so that these two com­pounds are far from being isomorphous. On the other hand, the third member of this group, com­pound (V), is monoclinic, so there can be no close similarities within this group.

None of the mol­ecules of (I)–(VIII) exhibits any inter­nal symmetry, so that they are all conformationally chiral; in each case, the reference mol­ecule was selected as one having a positive sign for the C3—C4—C41—C42 torsion angle (Table 2 ▸), although the space groups confirm that all the com­pounds have crystallized as conformational racemates.

**Figure 7:**
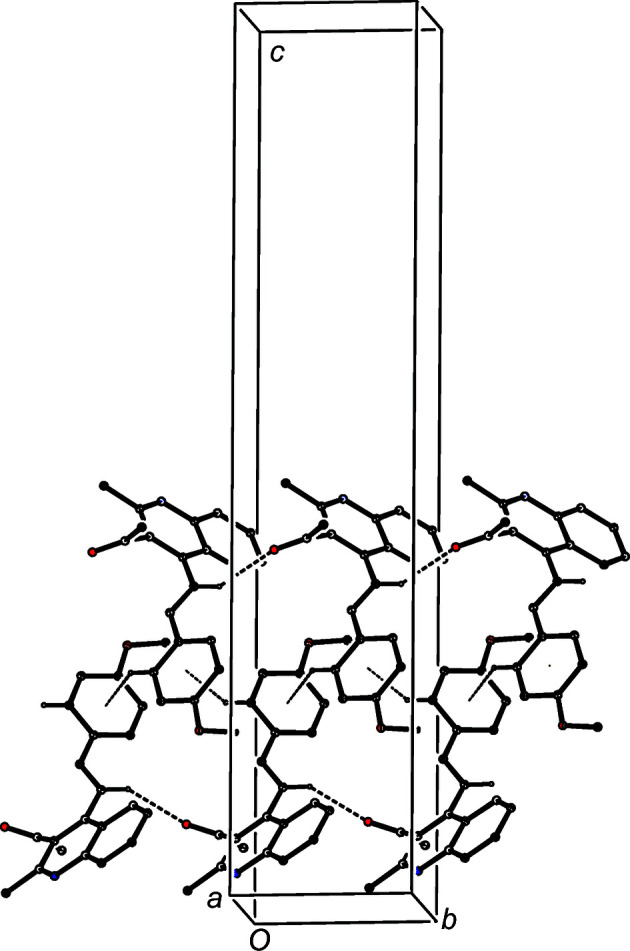
Part of the crystal structure of com­pound (I), showing the formation of a chain of rings along [010] built from C—H⋯O and C—H⋯π(arene) hydrogen bonds, drawn as dashed lines. For the sake of clarity, H atoms not involved in the motifs shown have been omitted.

The supra­molecular assembly of com­pounds (I)–(VI) is determined by C—H⋯O and C—H⋯π hydrogen bonds (Table 3 ▸). In each of (I)–(III), mol­ecules which are related by translation are linked by a C—H⋯O hydrogen bond to form a *C*(6) (Etter, 1990 ▸; Etter *et al.*, 1990 ▸; Bernstein *et al.*, 1995 ▸) chain running parallel to the [010] direction. In the structure of (I), this is enhanced by a C—H⋯π(arene) hydrogen bond linking mol­ecules related by the 2_1_ screw axis along (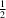, *y*, 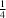) to form a chain of rings (Fig. 7 ▸). However, in the structures of (II) and (III), the corresponding H⋯*Cg* and C⋯*Cg* distances are much longer than they are in (I), so that these are possibly better regarded as short adventitious contacts rather than structurally significant hydrogen bonds.

**Figure 8:**
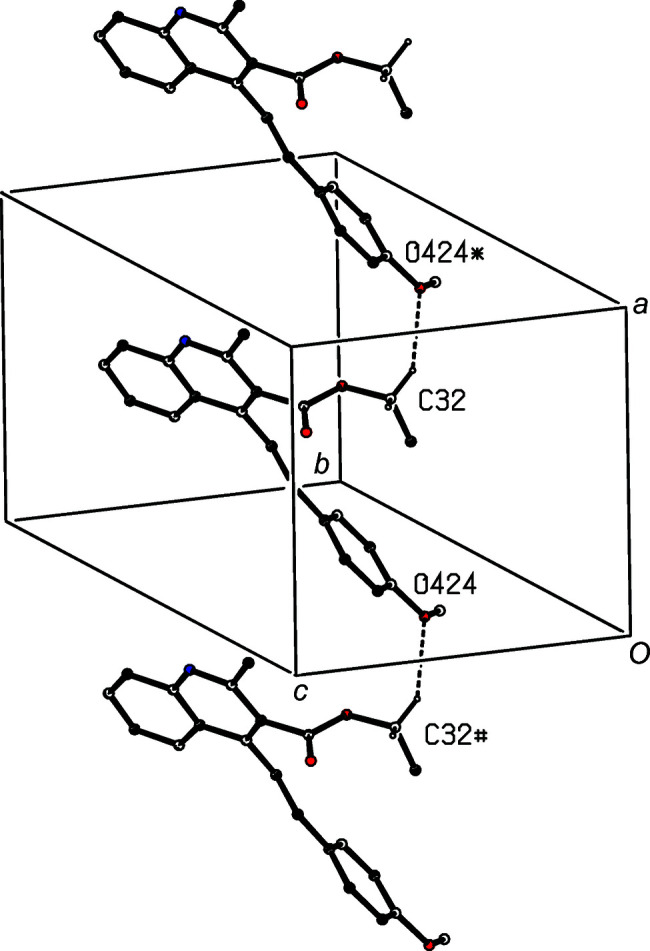
Part of the crystal structure of com­pound (IV), showing the formation of a *C*(13) chain running parallel to the [100] direction. Hydrogen bonds are drawn as dashed lines and, for the sake of clarity, H atoms bonded to those C atoms which are not involved in the motif shown have been omitted. Atoms marked with an asterisk (*) or a hash (#) are at the symmetry positions (*x* + 1, *y*, *z*) and (*x* − 1, *y*, *z*), respectively.

**Figure 9:**
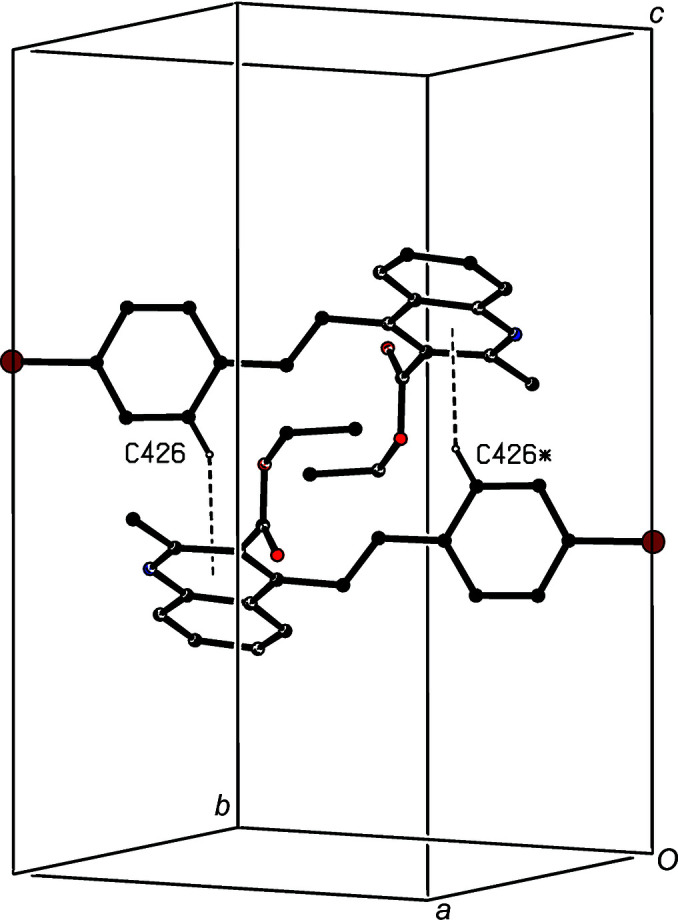
Part of the crystal structure of com­pound (V), showing the formation of a centrosymmetric dimer. Hydrogen bonds are drawn as dashed lines and, for the sake of clarity, H atoms which are not involved in the motif shown have been omitted. The atom marked with an asterisk (*) is at the symmetry position (−*x* + 1, −*y* + 1, −*z* + 1).

A single C—H⋯O hydrogen bond links the mol­ecules of com­pound (IV) which are related by translation into a *C*(13) chain running parallel to the [100] direction (Fig. 8 ▸). This structure also contains a short C—H⋯π(pyrid­yl) contact, but the long H⋯*Cg* distance and the very small C—H⋯*Cg* angle indicate that this is probably not structurally significant (Wood *et al.*, 2009 ▸). By contrast, in the structure of com­pound (V), it is the C—H⋯O contact which has a very small *D*—H⋯*A* angle (Table 3 ▸), while a C—H⋯π(pyrid­yl) hydrogen bond links mol­ecules which are related by inversion to form a cyclic centrosymmetric dimer (Fig. 9 ▸).

**Figure 10:**
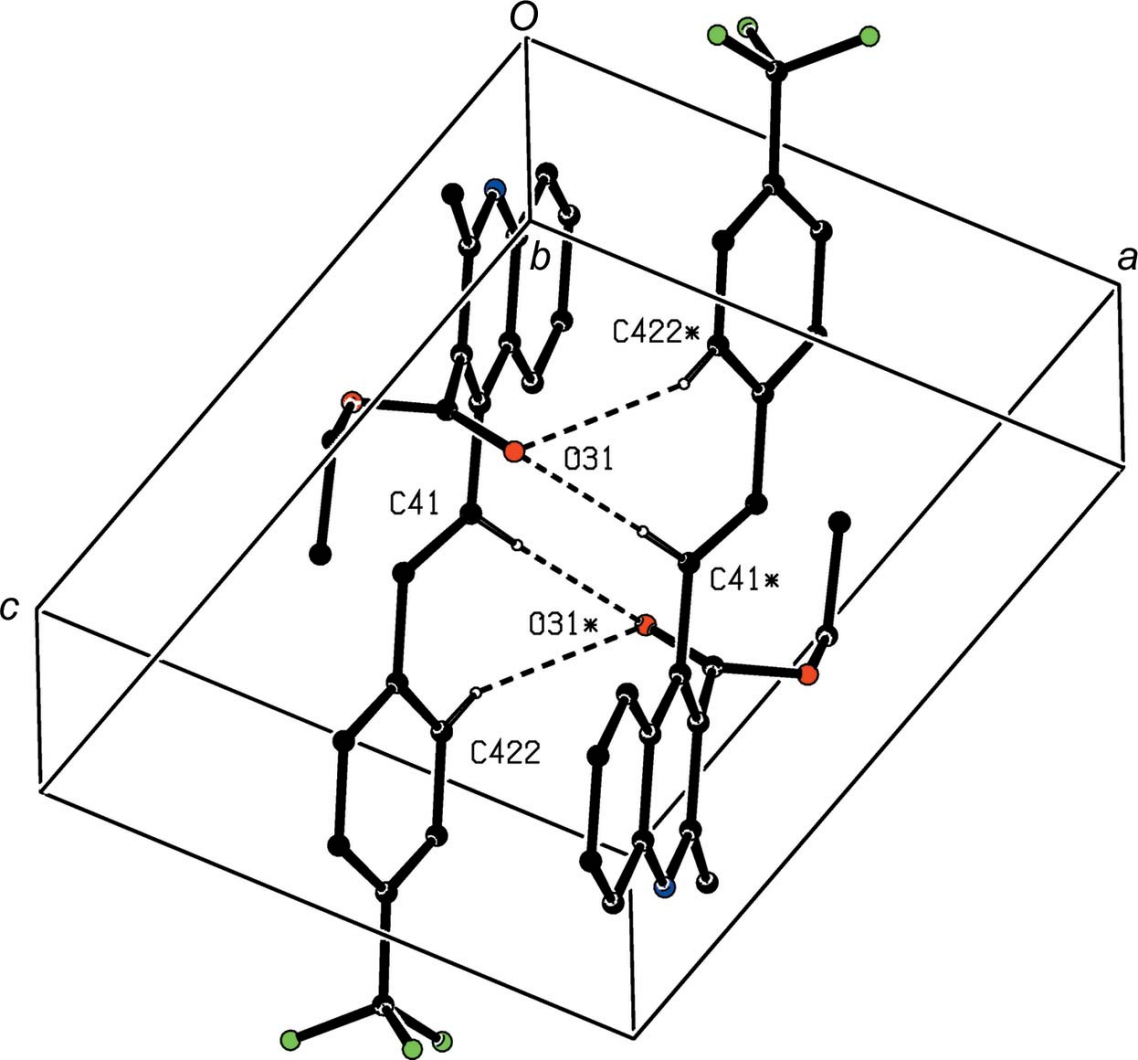
Part of the crystal structure of com­pound (VI), showing the formation of a centrosymmetric dimer. Hydrogen bonds are drawn as dashed lines and, for the sake of clarity, H atoms which are not involved in the motif shown have been omitted. Atoms marked with an asterisk (*) are at the symmetry position (−*x* + 1, −*y* + 1, −*z* + 1).

In the structure of com­pound (VI), there are no C—H⋯π hydrogen bonds or short inter­molecular contacts. Instead two C—H⋯O hydrogen bonds combine to link inversion-related pairs of mol­ecules into centrosymmetric dimers. The hydrogen bonds involving atoms of type C41 form an 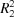
(12) ring, while those involving atoms of type C422 generate an 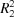
(18) ring (Fig. 10 ▸).

**Figure 11:**
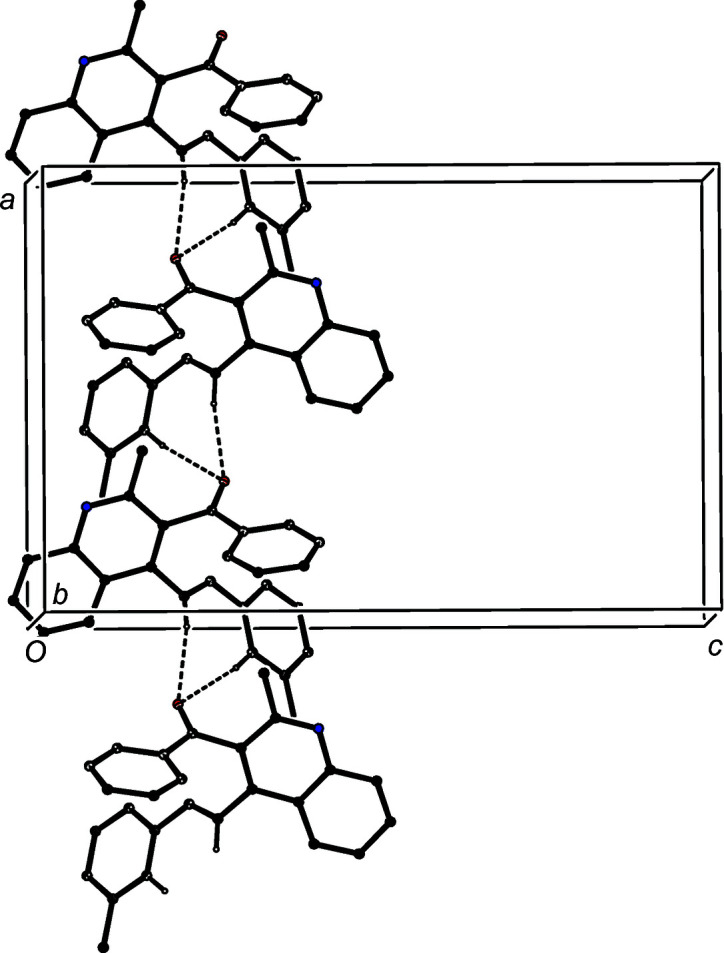
Part of the crystal structure of com­pound (VII), showing the formation of a *C*(6)*C*(9)[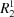
(7)] chain of rings running parallel to the [100] direction. Hydrogen bonds are drawn as dashed lines and, for the sake of clarity, H atoms which are not involved in the motif shown have been omitted.

**Figure 12:**
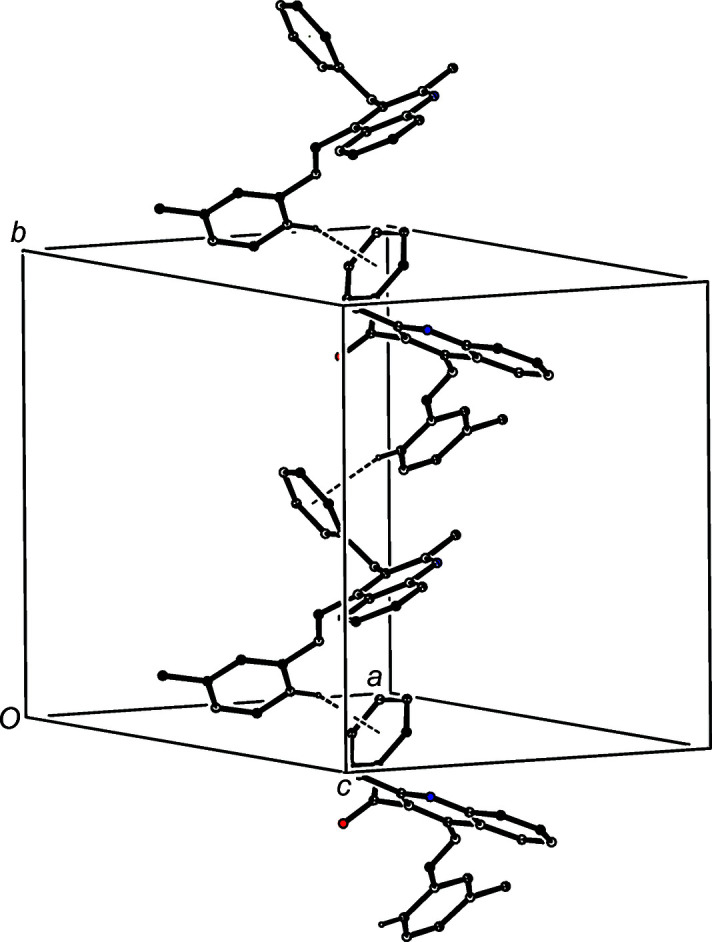
Part of the crystal structure of com­pound (VII), showing the formation of a chain built from C—H⋯π(arene) hydrogen bonds, drawn as dashed lines, running parallel to the [010] direction. For the sake of clarity, H atoms which are not involved in the motif shown have been omitted.

We also discuss here the supra­molecular assembly of com­pounds (VII) and (VIII), which, as noted above (§1, *Introduction*), have been reported on a simple proof of constitution basis, without discussion (Meléndez *et al.*, 2020 ▸). The assembly in (VII) in the space group *Pbcn* is based upon two C—H⋯O hydrogen bonds and one C—H⋯π(arene) hydrogen bond (Table 3 ▸). The two C—H⋯O hydrogen bonds link mol­ecules which are related by the *a*-glide plane at *z* = 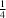 to form a *C*(6)*C*(9)[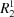
(7)] chain of rings running parallel to the [100] direction (Fig. 11 ▸). In addition, the structure of (VII) contains a C—H⋯π(arene) hydrogen bond which links mol­ecules which are related by the *b*-glide plane at *x* = 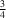 to form a chain running parallel to the [010] direction (Fig. 12 ▸). The combination of the chain motifs along [100] and [010] generates a com­plex sheet lying parallel to (001) in the domain 0 < *z* < 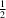; a second sheet of this type, related to the first by inversion, lies in the domain 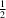 < *z* < 1.0, but there are no direction-specific inter­actions between adjacent sheets. Even the shortest inter­molecular contacts (Table 3 ▸) in chalcone (VIII) have H⋯*A* distances which are probably too long for these contacts to be regarded as structurally significant.

The structures of several simple 2-styryl­quino­lines have been published, including those of the unsubstituted 2-styryl­quino­line itself (Valle *et al.*, 1986 ▸), and of several analogues carrying simple substituents in the phenyl ring (Kuz’mina *et al.*, 2012 ▸). In addition, structures have been reported for a number of salts derived from 2-styryl­quino­lines (Kobkeatthawin *et al.*, 2008 ▸, 2009 ▸; Chantrapromma *et al.*, 2008 ▸, 2014 ▸; Fun *et al.*, 2013 ▸). For all of these com­pounds, the styryl group was introduced into a preformed quino­line nucleus. 8-Styryl­quino­line and its 4-phenyl­styryl analogue, whose structures have also been reported (Sharma *et al.*, 2015 ▸), were pre­pared using a rhodium-catalysed coupling reaction between quino­line *N*-oxide and the styrene com­ponent. Despite the substantial number of structure reports involving 2-styryl­quino­lines and their derivatives, there are no reports in the CSD of 4-styryl­quino­lines other than the two examples discussed above, *i.e.* com­pounds (VII) and (VIII) (CSD refcodes MUMZEC and MUMZIG, respectively; Meléndez *et al.*, 2020 ▸).

## Supplementary Material

Crystal structure: contains datablock(s) global, I, II, III, IV, V, VI. DOI: 10.1107/S2053229620010803/sk3755sup1.cif

Structure factors: contains datablock(s) I. DOI: 10.1107/S2053229620010803/sk3755Isup2.hkl

Structure factors: contains datablock(s) II. DOI: 10.1107/S2053229620010803/sk3755IIsup3.hkl

Structure factors: contains datablock(s) III. DOI: 10.1107/S2053229620010803/sk3755IIIsup4.hkl

Structure factors: contains datablock(s) IV. DOI: 10.1107/S2053229620010803/sk3755IVsup5.hkl

Structure factors: contains datablock(s) V. DOI: 10.1107/S2053229620010803/sk3755Vsup6.hkl

Structure factors: contains datablock(s) VI. DOI: 10.1107/S2053229620010803/sk3755VIsup7.hkl

Supporting information file. DOI: 10.1107/S2053229620010803/sk3755Isup8.cml

Supporting information file. DOI: 10.1107/S2053229620010803/sk3755IIsup9.cml

Supporting information file. DOI: 10.1107/S2053229620010803/sk3755IIIsup10.cml

Supporting information file. DOI: 10.1107/S2053229620010803/sk3755IVsup11.cml

Supporting information file. DOI: 10.1107/S2053229620010803/sk3755Vsup12.cml

Supporting information file. DOI: 10.1107/S2053229620010803/sk3755VIsup13.cml

Crystal structure: contains datablock(s) VII, VIII. DOI: 10.1107/S2053229620010803/sk3755sup14.cif

CCDC references: 2021528, 2021527, 2021526, 2021525, 2021524, 2021523

## Figures and Tables

**Figure 2:**
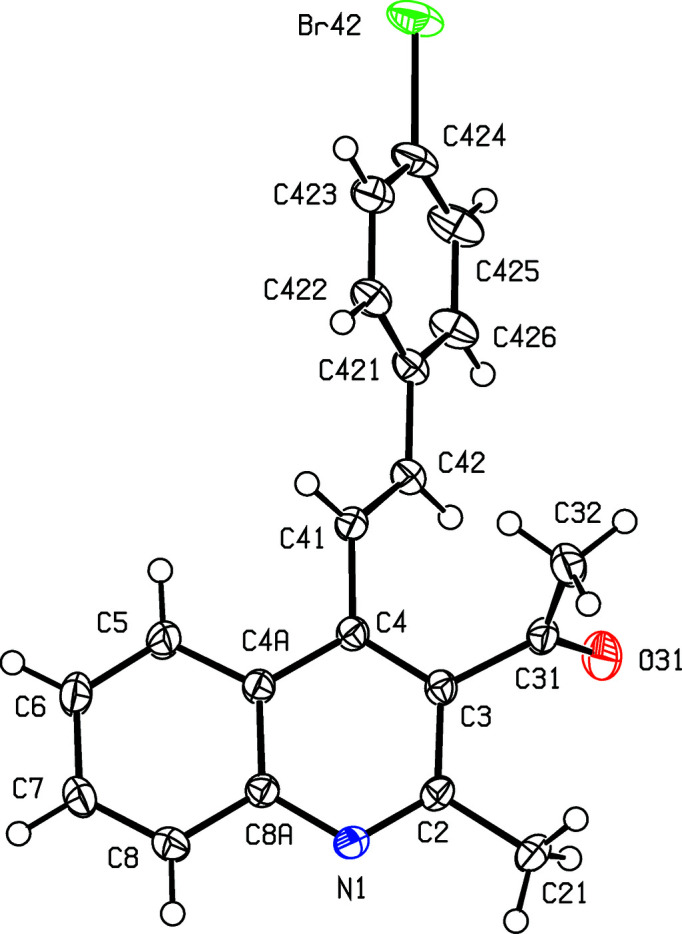
The mol­ecular structure of com­pound (II), showing the atom-labelling scheme. Displacement ellipsoids are drawn at the 50% probability level.

**Figure 3:**
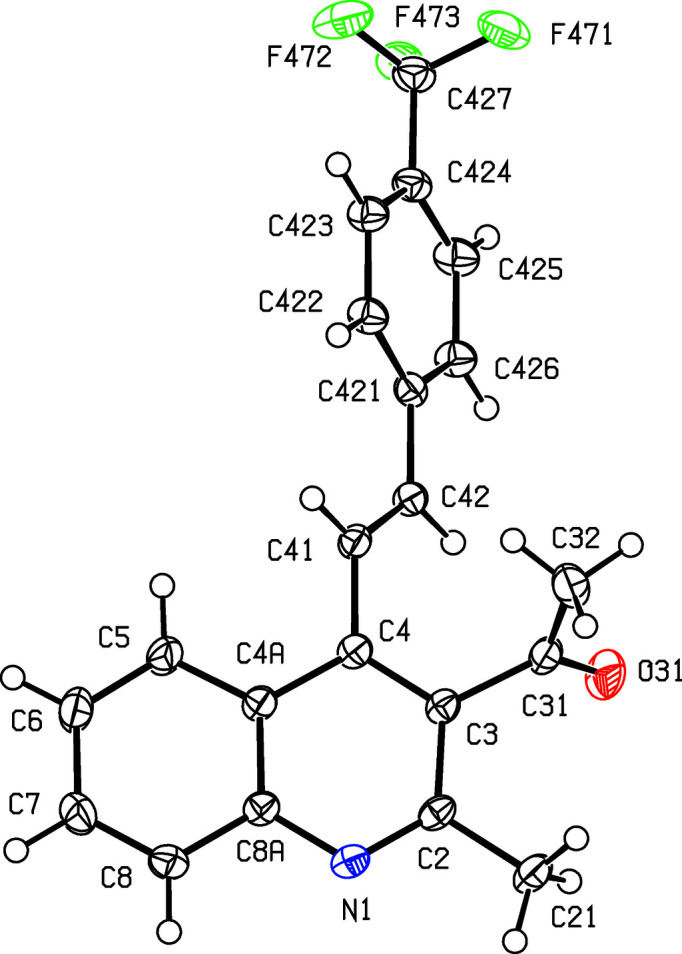
The mol­ecular structure of com­pound (III), showing the atom-labelling scheme. Displacement ellipsoids are drawn at the 50% probability level.

**Figure 5:**
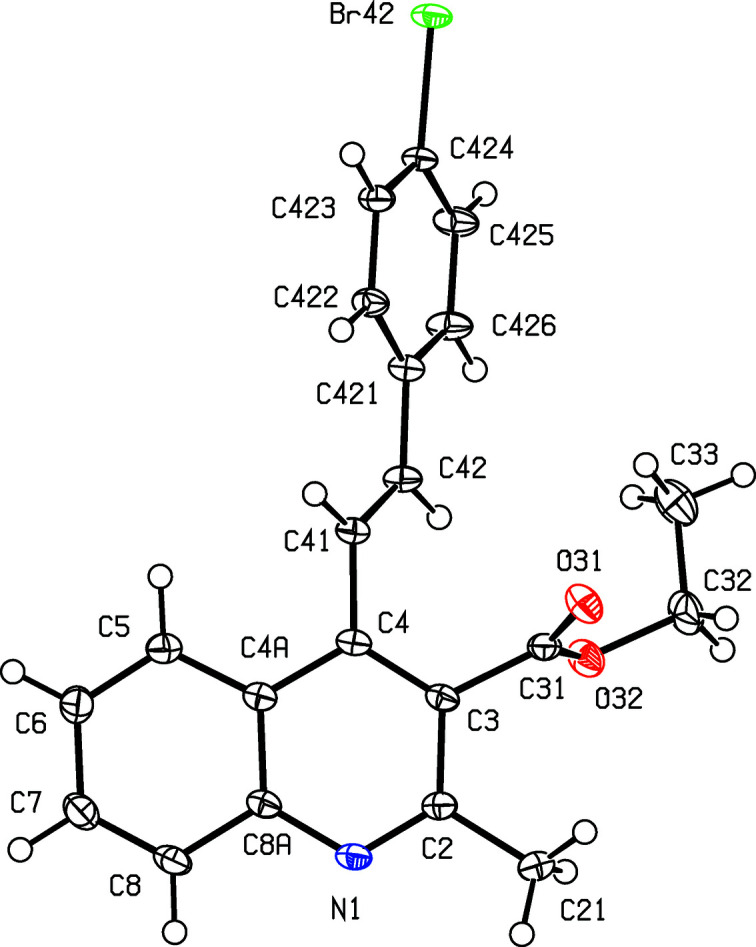
The mol­ecular structure of com­pound (V), showing the atom-labelling scheme. Displacement ellipsoids are drawn at the 50% probability level.

**Figure 6:**
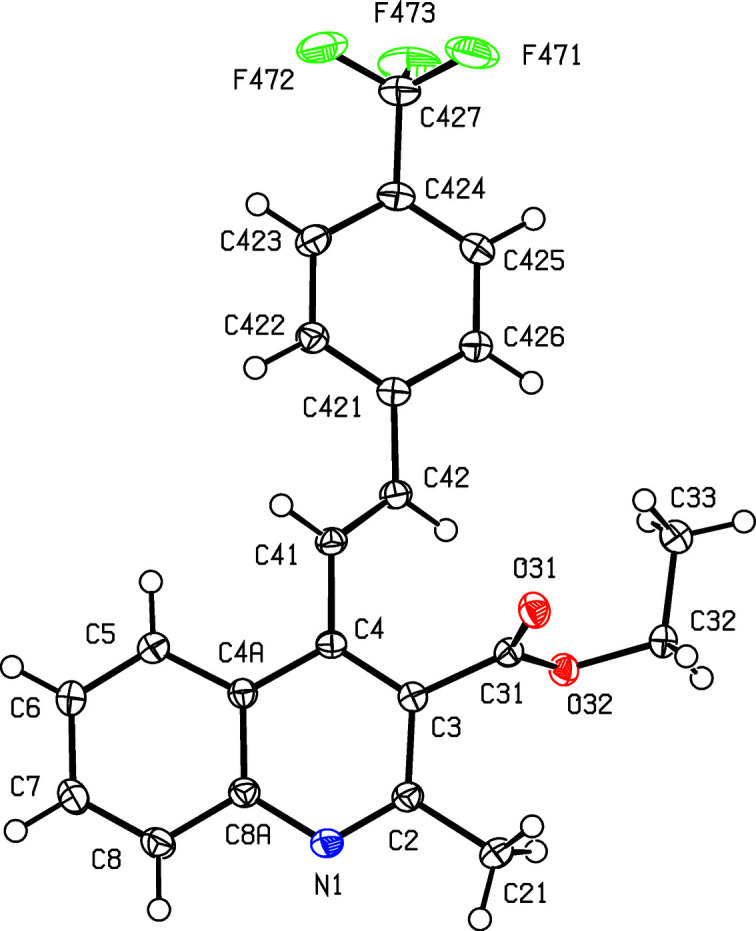
The mol­ecular structure of com­pound (VI), showing the atom-labelling scheme. Displacement ellipsoids are drawn at the 50% probability level.

**Table:** Experiments were carried out at 100 K with Mo *K*α radiation using a Bruker D8 Venture diffractometer. Absorption was corrected for by multi-scan methods (*SADABS*; Bruker, 2016 ▸). H-atom parameters were constrained.

	(I)	(II)	(III)
Crystal data			
Chemical formula	C_21_H_19_NO_2_	C_20_H_16_BrNO	C_21_H_16_F_3_NO
*M*_r_	317.37	366.24	355.35
Crystal system, space group	Monoclinic, *P*2_1_/*c*	Monoclinic, *P*2_1_/*c*	Monoclinic, *P*2_1_/*c*
*a*, *b*, *c* (Å)	8.2595 (4), 6.4279 (3), 31.9064 (14)	8.0849 (3), 6.6692 (2), 31.1063 (10)	8.0822 (4), 6.6567 (4), 32.1024 (17)
α, β, γ (°)	90, 93.674 (2), 90	90, 95.005 (1), 90	90, 90.576 (2), 90
*V* (Å^3^)	1690.47 (14)	1670.85 (10)	1727.05 (16)
*Z*	4	4	4
μ (mm^−1^)	0.08	2.46	0.11
Crystal size (mm)	0.23 × 0.16 × 0.09	0.17 × 0.11 × 0.04	0.20 × 0.08 × 0.06

Data collection			
*T*_min_, *T*_max_	0.947, 0.993	0.810, 0.906	0.942, 0.994
No. of measured, independent and observed [*I* > 2σ(*I*)] reflections	39800, 4197, 3528	38342, 3839, 3434	19648, 3959, 3363
*R*_int_	0.051	0.034	0.032
(sin θ/λ)_max_ (Å^−1^)	0.668	0.650	0.650

Refinement			
*R*[*F* ^2^ > 2σ(*F* ^2^)], *wR*(*F* ^2^), *S*	0.040, 0.111, 1.06	0.030, 0.073, 1.03	0.041, 0.101, 1.04
No. of reflections	4197	3839	3959
No. of parameters	220	210	237
Δρ_max_, Δρ_min_ (e Å^−3^)	0.33, −0.23	0.56, −0.76	0.28, −0.23

**Table:** 

	(IV)	(V)	(VI)
Crystal data			
Chemical formula	C_22_H_21_NO_3_	C_21_H_18_BrNO_2_	C_22_H_18_F_3_NO_2_
*M*_r_	347.40	396.26	385.37
Crystal system, space group	Triclinic, *P* 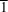	Monoclinic, *P*2_1_/*n*	Triclinic, *P* 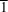
*a*, *b*, *c* (Å)	9.5301 (8), 10.3513 (8), 10.3621 (8)	9.5709 (6), 10.6119 (7), 18.2074 (10)	8.7465 (10), 9.9436 (11), 11.1116 (11)
α, β, γ (°)	65.374 (3), 86.583 (3), 76.376 (3)	90, 90.939 (2), 90	105.446 (4), 99.763 (4), 97.204 (4)
*V* (Å^3^)	902.23 (13)	1849.0 (2)	903.08 (17)
*Z*	2	4	2
μ (mm^−1^)	0.09	2.24	0.11
Crystal size (mm)	0.30 × 0.12 × 0.05	0.25 × 0.18 × 0.15	0.27 × 0.20 × 0.18

Data collection			
*T*_min_, *T*_max_	0.954, 0.996	0.595, 0.715	0.954, 0.980
No. of measured, independent and observed [*I* > 2σ(*I*)] reflections	44489, 4158, 3440	54534, 4588, 4098	59876, 4491, 3692
*R*_int_	0.053	0.041	0.042
(sin θ/λ)_max_ (Å^−1^)	0.650	0.667	0.667

Refinement			
*R*[*F* ^2^ > 2σ(*F* ^2^)], *wR*(*F* ^2^), *S*	0.052, 0.128, 1.07	0.022, 0.056, 1.02	0.044, 0.120, 1.05
No. of reflections	4158	4588	4491
No. of parameters	238	228	255
Δρ_max_, Δρ_min_ (e Å^−3^)	0.36, −0.21	0.35, −0.42	0.43, −0.43

Computer programs: *APEX3* (Bruker, 2018 ▸), *SAINT* (Bruker, 2017 ▸), *SHELXT2014* (Sheldrick, 2015*a* ▸), *SHELXL2014* (Sheldrick, 2015*b* ▸) and *PLATON* (Spek, 2020 ▸).

**Table 2:** Selected torsion angles (°) for com­pounds (I)–(VIII)

Compound	C3—C4—C41—C42	C41—C42—C421—C422	C2—C3—C31—O31	C2—C3—C31—O32
(I)	46.40 (16)	13.78 (18)	66.18 (15)	
(II)	46.8 (3)	14.8 (3)	68.2 (2)	
(III)	49.0 (2)	13.0 (2)	69.52 (18)	
(IV)	48.8 (2)	1.2 (3)	73.7 (2)	−104.85 (17)
(V)	51.4 (2)	10.6 (2)	−102.56 (17)	75.88 (15)
(VI)	34.8 (2)	−17.7 (2)	−99.75 (16)	76.46 (15)
(VII)	51.0 (5)	2.0 (5)	71.5 (4)	
(VIII)	54.17 (19)	−4.0 (2)	−97.59 (15)	

**Table 3:** Hydrogen bonds and short intra­molecular contacts (Å, °) for com­pounds (I)–(VIII) *Cg*1, *Cg*3, *Cg*4 and *Cg*5 represent the centroids of the N1/C2–C4/C4*A*/C8*A*, C421–C426, C311–C316 [present in (VII) only] and C331–C336 [present in (VIII) only] rings, respectively; ring 2 com­prises atoms C4*A*/C5–C8/C8*A*.

	*D*—H⋯*A*	*D*—H	H⋯*A*	*D*⋯*A*	*D*—H⋯*A*
(I)	C41—H41⋯O31^i^	0.95	2.41	3.2527 (15)	148
	C426—H426⋯*Cg*3^ii^	0.95	2.77	3.5252 (13)	138
(II)	C41—H41⋯O31^i^	0.95	2.37	3.283 (2)	161
	C426—H426⋯*Cg*3^ii^	0.95	2.91	3.7071 (19)	142
(III)	C41—H41⋯O31^i^	0.95	2.42	3.3290 (17)	161
	C426—H426⋯*Cg*3^ii^	0.95	3.00	3.7621 (15)	138
(IV)	C32—H32*B*⋯O424^iii^	0.99	2.57	3.406 (2)	142
	C423—H423⋯*Cg*1^iv^	0.95	2.91	3.4894 (19)	120
(V)	C423—H423⋯O31^v^	0.95	2.59	3.2197 (17)	124
	C426—H426⋯*Cg*1^iv^	0.95	2.74	3.5961 (16)	151
(VI)	C41—H41⋯O31^iv^	0.95	2.54	3.4922 (18)	178
	C422—H422⋯O31^iv^	0.95	2.56	3.3924 (19)	146
(VII)	C41—H41⋯O31^vi^	0.95	2.37	3.300 (5)	167
	C422—H422⋯O31^vi^	0.95	2.57	3.506 (4)	169
	C426—H426⋯*Cg*4^vii^	0.95	2.67	3.549 (4)	155
(VIII)	C334—H334⋯O31^viii^	0.95	2.61	3.542 (2)	168
	C7—H7⋯*Cg*5^ix^	0.95	2.93	3.6437 (16)	133
	C422—H422⋯*Cg*1^x^	0.95	2.93	3.6300 (17)	132

Symmetry codes: (i) *x*, *y* + 1, *z*; (ii) −*x* + 1, *y* − 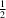, −*z* + 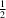; (iii) *x* + 1, *y*, *z*; (iv) −*x* + 1, −*y* + 1, −*z* + 1; (v) −*x* + 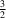, *y* + 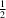, −*z* + 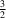; (vi) *x* − 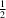, *y*, −*z* + 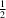; (vii) −*x* + 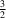, *y* − 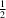, *z*; (viii) *x* − 1, *y*, *z*; (ix) *x* + 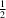, −*y* + 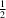, *z* − 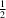; (x) *x* + 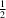, −*y* + 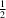, *z* + 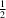.
